# Could Ocular Glands Be Infected by SARS-CoV-2?

**DOI:** 10.3390/diseases12080169

**Published:** 2024-07-25

**Authors:** Jinghua Bu, Minjie Zhang, Rongrong Zhang, Le Sun, Zhenzong Chen, Yang Wu

**Affiliations:** 1 Zhongshan Hospital (Xiamen), Fudan University, Xiamen 361006, China; 2 Department of Ophthalmology, Xiang’an Hospital of Xiamen University, Fujian Provincial Key Laboratory of Ophthalmology and Visual Science, Eye Institute of Xiamen University, School of Medicine, Xiamen University, Xiamen 361102, China; jhbu@xah.xmu.edu.cn (J.B.); minjiezhang@xmu.edu.cn (M.Z.); rongrongzhang1998@stu.xmu.edu.cn (R.Z.); sunle777@stu.xmu.edu.cn (L.S.)

**Keywords:** ACE2, SARS-CoV-2, Meibomian gland, ocular gland, inflammation

## Abstract

The aim of the study was to investigate the expression levels of ACE2 in ocular glands and to investigate the effect of S protein on them. Male C57BL/6J mice were used for the experiments. The expression levels of ACE2 are highest in the Meibomian glands, followed by the conjunctiva, the cornea, and the lacrimal glands. Co-immunoprecipitation assays confirmed direct binding between ACE2 and S protein in ocular surface epithelia and Meibomian glands. CD45+ cell infiltration was found in the S protein treatment group, which was accompanied by upregulation of inflammation-related cytokines. There was also prominent cell apoptosis in the S protein treatment group. In conclusion, not only the cornea and the conjunctiva, but also the Meibomian glands express ACE2, and S protein could induce ocular surface epithelial cell and Meibomian gland cell inflammation and apoptosis.

## 1. Introduction

Coronavirus disease 2019 (COVID-19), caused by severe acute respiratory syndrome coronavirus 2 (SARS-CoV-2), has infected billions of people around the world [1]. Patients with COVID-19 show typical respiratory symptoms, including cough, fever, and lung damage, and other symptoms, such as fatigue, myalgia, and diarrhea [2]. How SARS-CoV-2 is transmitted is not fully understood. SARS-CoV-2 spreads mainly through respiratory secretions or droplets [3]. A recent study showed that aerosols containing SARS-CoV-2 virions remained infectious during a 3 h period of observation [4]. SARS-CoV-2 RNA has been detected in the stools, whole blood, and urine of COVID-19 patients, but it is unclear whether it can be transmitted through these media [5]. SARS-CoV-2 was also found in conjunctivae of COVID-19 patients by RT-qPCR [6], indicating that the ocular surface may be an entry point through exposure to respiratory droplets or hand–eye contact. Another study confirmed that one-third of COVID-19 patients had ocular manifestations, which frequently occurred in more severe COVID-19 patients [7,8], suggesting that the SARS-CoV-2 virus could induce ocular surface damage.

SARS-CoV-2 belongs to the β coronavirus genus, like SARS-CoV and MERS-CoV [9]. The structural proteins of SARS-CoV-2 include the spike (S) protein, the envelope (E) protein, the membrane (M) protein, and the nucleocapsid (N) protein [10]. The S protein is responsible for attachment to the host receptor and fusion with the cell membrane [11]. The E protein contributes to the assembly of virus particles [12]. The M protein helps in the assembly of new virus particles [13]. The N protein interacts with viral RNA to form ribonucleoprotein [14].

Numerous studies have shown that S protein plays a key role in receptor binding for the entry of SARS-CoV-2 into target cells [15,16]. Angiotensin-converting enzyme 2 (ACE2), as the cellular receptor, binds to the receptor-binding domain (RBD) of the SARS-CoV-2 S protein, which is the critical step for the virus to enter target cells [15]. This mechanism for viral entry is also used by SARS-CoV [17]. Two recent studies have confirmed the expression of ACE2 in human ocular surface epithelia, including the cornea and the conjunctiva [18,19]. However, it is unclear whether SARS-CoV-2 could affect the ocular glands, including the Meibomian glands, which secrete meibum, which forms the outer layer of the tear film, and the lacrimal glands, which produce the aqueous component of the tear film. In addition, although we have known about the expression of ACE2 in the cornea and the conjunctiva, we still do not know whether S protein can bind to ACE2 in the ocular surface and then induce damage to the ocular surface.

In the current study, we found that the Meibomian glands strongly express ACE2 at a higher level than the cornea and the conjunctiva. We also confirmed that S protein can bind to ACE2 in ocular surface epithelia and ocular glands, leading to inflammation and apoptosis of ocular surface cells. Taken together, these results suggest that not only ocular surface epithelia but also Meibomian glands express ACE2 and that they are all susceptible to affection by S protein. The lacrimal glands seem not to be susceptible to SARS-CoV-2.

## 2. Materials and Methods

### 2.1. Animals

Male C57BL/6J (B6) mice aged 8 to 12 weeks old were purchased from the Shanghai SLAC Laboratory Animal Center. The animals were housed at the Experimental Animal Center of Xiamen University. The research protocol followed the standards of the ARVO Statement on the use of animals and was approved by the Experimental Animal Ethics Committee of Xiamen University. To evaluate the effect of the SARS-CoV-2 spike (S) protein (catalog no. 40591-V08H; Sino Biological, Beijing, China) and the nucleocapsid (N) protein (catalog no. 40588-V07E; Sino Biological, Beijing, China) on the ocular surface, topical S and N protein treatments were performed on B6 mice by the application of 2 μL 20 ng/mL S and N protein or vehicle (PBS) eye drops twice daily for 7 days.

### 2.2. Western Blot Analysis

After mouse sacrifice, we cut along the limbus and the lower margin of the tarsal plate to obtain the majority of the conjunctiva. Eyes were hemisected along the boundary separating the white conjunctiva and the clear cornea. Then, the lens, the iris, and as much of the ciliary body as possible were removed from the cornea. The Meibomian glands were isolated from eyelids under a dissecting microscope by removing the skin, subcutaneous tissue, muscle, and palpebral conjunctiva. Lacrimal glands were dissected from the anterior and ventral to the ear. The corneal epithelium, conjunctival, lacrimal gland, and Meibomian gland proteins were extracted using RIPA Lysis Buffer containing protease and phosphatase inhibitor. A BCA protein assay kit (catalog no. 23225; Thermo Fisher Scientific, Waltham, MA, USA) was used to determine the total protein concentration. Equal amounts of proteins were loaded into the wells of SDS-PAGE gel and left for 1 h under a voltage of 110 V. Subsequently, the separated proteins were transferred to polyvinylidene difluoride (PVDF) membranes (catalog no. IPVH00010; Millipore, Bedford, MA, USA). After blocking in 2% BSA for 1 h, the membranes were incubated overnight in anti-ACE2 primary antibody (1:500; catalog no. 11802-1-AP; Proteintech, Chicago, IL, USA) and β-actin (1:10,000; catalog no. A3854; Sigma-Aldrich, St. Louis, MO, USA) solutions at 4 °C. The antibodies were diluted in TBST and 5% nonfat dried milk at the manufacturer’s recommended dilution. The blots were rinsed 3 times for 5 min with Tris-buffered saline with 0.05% Tween 20. Next, the blots were incubated with horseradish peroxidase (HRP)-conjugated goat anti-rabbit IgG (1:10,000; catalog no. a0545; Sigma-Aldrich, USA) for 1 h at room temperature. The results were visualized using enhanced chemiluminescence reagents (catalog no. ECL-500; ECL, Lulong, Inc., Xiamen, China). Chemiluminescent signals were captured and analyzed by a transilluminator (ChemiDoc XRS System; Bio-Rad Laboratories, Hercules, CA, USA).

### 2.3. Co-Immunoprecipitation

Corneal epithelium, conjunctival, lacrimal gland, and Meibomian gland proteins were extracted and measured as described above. Quantities of 1 μg Anti-His antibody (catalog no. 66005-1-Ig; Sino Biological, China) and 1 μg S protein with His tag (catalog no. 40591-C08H; Sino Biological, China) were added to the tissue lysate. Then, the mix was incubated overnight at 4 °C with gentle agitation to form the immunocomplex, followed by incubation with Pierce Protein A/G Magnetic Beads (catalog no. 23225; Thermo Fisher Scientific, USA). After microcentrifugation for 30 s at 4 °C, the beads were collected and washed five times with cell lysis buffer on ice. Subsequently, the beads were collected and heated to 95–100 °C for 5 min with 1× protein loading buffer. Finally, the samples were loaded on SDS-PAGE gel (12–15%) and analyzed by Western blotting.

### 2.4. Immunofluorescence Staining

Frozen sections (6 μm in thickness) of mouse ocular tissue were fixed with cold acetone for 10 min. After washing them 3 times with PBS, the sections were incubated with 0.2% Triton X-100 for 20 min. The sections were blocked in 2% BSA for 1 h at room temperature, followed by incubation with anti-ACE2 (1:200; catalog no. 21115-1-AP; Protech Co., Wuhan, China) and CD45 (1:200; catalog no. sc-52491; Santa Cruz Biotechnology, Santa Cruz, CA, USA) primary antibodies at 4 °C overnight. Subsequent to incubation, the slices were washed three times with PBS, then stained with AlexaFluor 488-conjugated donkey anti-rabbit, -mouse, or -rat IgG as the second antibody. After three additional washes for 10 min each with PBS, the slides were sealed with glycerol with DAPI and then observed under a Leica upright microscope (DM2500; Leica Microsystems, Wetzlar, Germany).

### 2.5. TUNEL Assay

TUNEL staining was performed using a commercially available kit (DeadEnd Fluorometric TUNEL System; catalog no. G3250; Promega, Madison, WI, USA) on paraffin-embedded sections. TUNEL assays were conducted according to the kit manufacturer’s instructions. Briefly, the frozen sections were fixed by 4% paraformaldehyde and incubated with Proteinase K Tris/HCL (pH 7.4) (10 mmol/L) for 30 min at 37 °C. Then, 50 mL of terminal deoxynucleotidyl transferase-mediated dUTP nick-end labeling reaction mixture was added and incubated for 1 h at 37 °C in the dark. The sections were mounted using medium with DAPI. Picture acquisition was performed using a Leica upright microscope (DM2500; Leica Microsystems, Wetzlar, Germany).

### 2.6. RNA Extraction and RT-qPCR

Total RNA was isolated from the samples using the RNeasy Micro kit (catalog no. 74004; QIAGEN, Duesseldorf, Germany). Samples were reversed transcribed to cDNA using the ExScript RT Reagent kit (catalog no. DRR035A; Takara, Tokyo, Japan). Further, the cDNA products were processed by qPCR using the SYBR Premix Ex Taq Kit (catalog no. RR420A; Takara, Tokyo, Japan). The qPCR reaction program was as follows: pre-incubation at 95 °C for 10 min followed by amplification at 95 °C for 10 s, 57 °C for 30 s, and 75 °C for 10 s, for 40 cycles. All primers for qPCR were designed using the Primer 3 system and were verified to produce a single peak in the melting curve using the StepOne Real-Time PCR detection system (Applied Biosystems, Thermo Fisher Scientific, Waltham, MA, USA). In relative quantitative real-time PCR, threshold numbers (Ct values) were set within the exponential phase of the reaction, and the relative expression for each gene was normalized to β-actin expression.

### 2.7. Statistical Analysis

Statistical analysis was performed by one-way ANOVA with Tukey’s post hoc test using GraphPad Prism 6.0 software (GraphPad Software, Inc., San Diego, CA, USA). Summary data are represented as means ± SDs. *p* < 0.05 was considered statistically significant.

## 3. Results

### 3.1. Expression of ACE2 in Ocular Surface Epithelia and Ocular Glands

**Figure 1:**
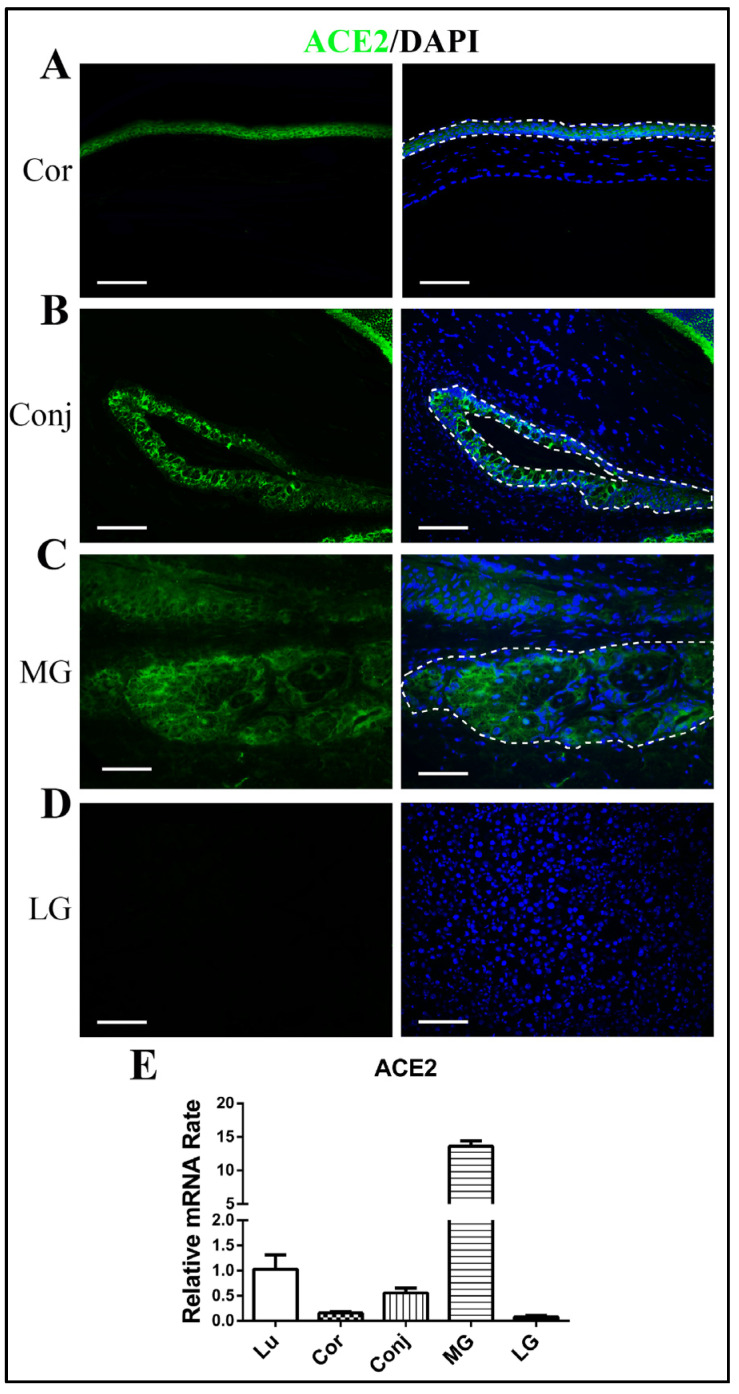
Expression of ACE2 in ocular surface epithelia and ocular glands. Immunofluorescence staining showing the expression of ACE2 in the cornea (**A**), the conjunctiva (**B**), Meibomian glands (**C**), and lacrimal glands (**D**). Quantitative RT-qPCR showing the gene expression of ACE2 in lung tissue, the cornea, the conjunctiva, Meibomian glands, and lacrimal glands (**E**). Scale bars: 150 μm. n = 6. Lu, lung; Cor, cornea; Conj, conjunctiva; MG, Meibomian gland; LG, lacrimal gland.

Immunofluorescence staining of ACE2 revealed positive staining in corneal epithelia and conjunctival epithelia, and there was negative expression of ACE2 in the corneal stroma and conjunctiva goblet cells (Figure 1A,B). Meibomian glands also showed clear positive staining of ACE2, mainly in the acinar cells, but there was very little staining in lacrimal glands (Figure 1C,D). The RT-qPCR results confirmed the higher expression of ACE2 in the Meibomian glands than in the corneal epithelium and the conjunctival epithelium—even higher than in lung tissue (Figure 1E). The expression of ACE2 in the lacrimal glands was very low (Figure 1E).

### 3.2. Direct Binding of S Protein to ACE2 in Ocular Surface Epithelia and Meibomian Glands

**Figure 2:**
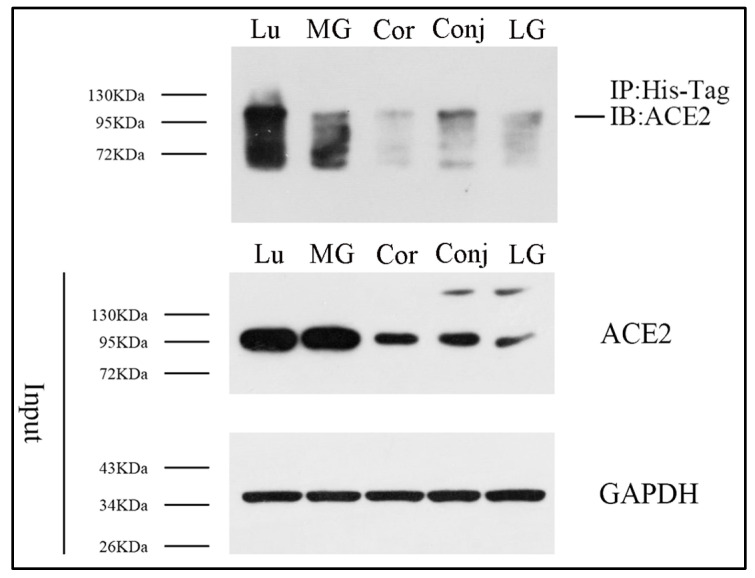
S protein can bind with ACE2 in ocular surface epithelia and Meibomian glands. Co-immunoprecipitation analysis was performed to detect the potential binding between S protein and ACE2 in lung tissue, the cornea, the conjunctiva, Meibomian glands, and lacrimal glands. n = 3. Lu, lung; Cor, cornea; Conj, conjunctiva; MG, Meibomian gland; LG, lacrimal gland.

In order to find if S protein binds directly to ACE2 in ocular surface epithelia and Meibomian glands, we performed co-immunoprecipitation assays. Following immunoprecipitation of S protein with its ACE2 antibodies, ACE2 was detected by Western blotting in the immunoprecipitated material (Figure 2). Our co-immunoprecipitation data evidently suggested that S protein bound with ACE2 in ocular surface epithelia and Meibomian glands in vitro. The expression level of ACE2 in Meibomian glands was much higher than in ocular surface epithelia and lacrimal glands, and the binding between ACE2 and S protein in Meibomian glands was shown to be stronger than in ocular surface epithelia and lacrimal glands. The binding between ACE2 and S protein increased with ACE2 expression, suggesting that Meibomian glands are susceptible to the virus.

### 3.3. S Protein Induces Inflammation-Related Cytokine Upregulation in Ocular Surface Epithelia and Meibomian Glands

**Figure 3:**
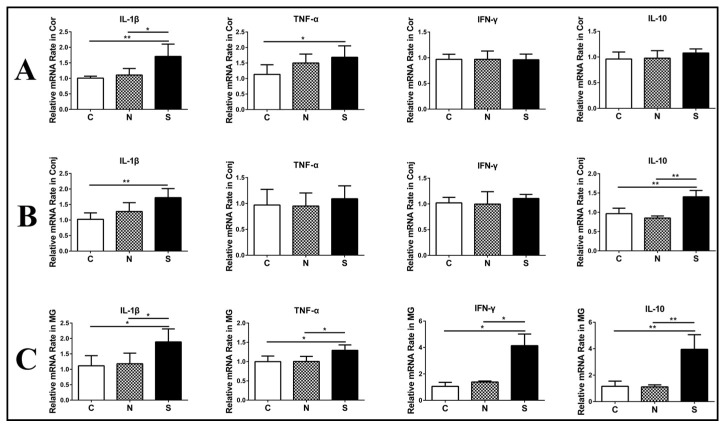
S protein induces inflammation-related cytokine upregulation in ocular surface epithelia and Meibomian glands. RT-qPCR showing the gene expression of IL-1β, TNF-α, INF-γ, and IL-10 in the cornea (**A**), the conjunctiva (**B**), and Meibomian glands (**C**). C, control group; N, N protein-treated group; S, S protein-treated group. Data are shown as means ± SDs; n = 6. * *p* < 0.05, ** *p* < 0.01.

Several studies have reported that SARS-CoV-2 S protein can induce inflammatory cytokine and chemokine expression in lung epithelial cells [20,21]. However, it is poorly understood whether SARS-CoV-2 S protein can induce ocular surface epithelial cells to produce proinflammatory cytokines. To address this concern, we stimulated mouse eyes with S protein. In the cornea, IL-1β and TNF-α mRNA levels were generally higher after S protein treatment, but no significant difference in IFN-γ or IL-10 mRNA levels was observed (Figure 3A). In the conjunctiva, IL-1β and IL-10 mRNA levels obviously increased after S protein treatment, but there was no significant difference in IFN-γ or TNF-α mRNA levels (Figure 3B). In the Meibomian glands, IL-1β, TNF-α, IFN-γ and IL-10 mRNA levels significantly increased after S protein treatment (Figure 3C). In addition, N protein could not induce inflammation in ocular surface epithelia and Meibomian glands (Figure 3A–C).

### 3.4. S Protein Induces Inflammatory Cell Infiltration in Ocular Surface Epithelia and Meibomian Glands

**Figure 4:**
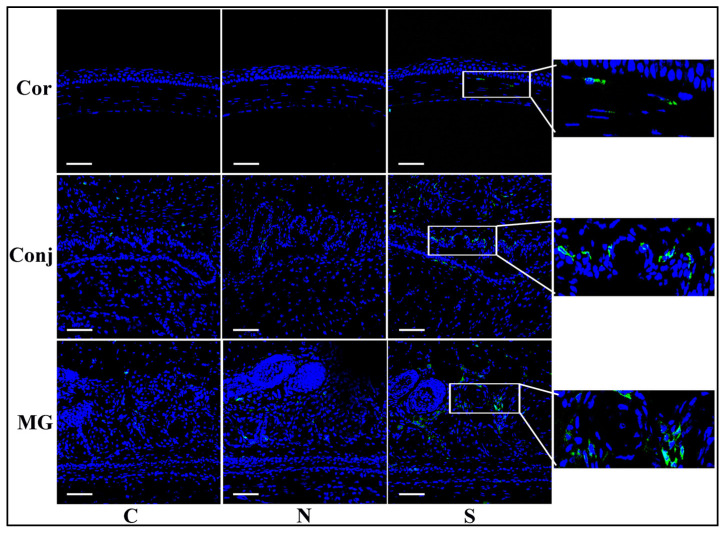
S protein induces inflammatory cell infiltration in ocular surface epithelia and Meibomian glands. Immunofluorescent staining of CD45 showing significantly more staining in the S protein-treated group than in the N protein-treated group in the cornea, the conjunctiva, and the Meibomian glands. Scale bars: 100 μm. n = 6.

CD45 immunofluorescent staining showed more positive cells in the S protein treatment group than in the N protein treatment group and the control group in the cornea, the conjunctiva, and the Meibomian glands (Figure 4). These results indicated that more immune cells infiltrated the cornea, the conjunctiva, and the Meibomian glands after S protein treatment.

### 3.5. S Protein Promotes Cell Apoptosis in Ocular Surface Epithelia and Meibomian Glands

**Figure 5:**
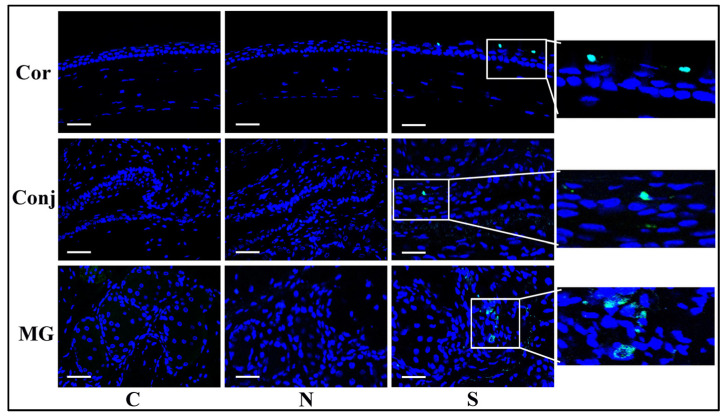
S protein promotes cell apoptosis in ocular surface epithelia and Meibomian glands. TUNEL staining showing significantly more staining in the S protein-treated group than in the N protein-treated group in the cornea, the conjunctiva, and the Meibomian glands. Scale bars: 100 μm. n = 6.

S protein induced marked TUNEL staining in the corneal and conjunctival epithelial cells and Meibomian gland acinar cells, which was scant in the N protein treatment group and the control group (Figure 5). These results indicated that S protein induced apoptosis in the cornea, the conjunctiva, and the Meibomian glands.

## 4. Discussion

SARS-CoV-2 is spreading more rapidly across the globe than SARS-CoV did, and there is a large amount of asymptomatic transmission [3]. Therefore, transmissions must be prevented in order to stop the rapidly increasing trend. However, currently, the transmission routes of SARS-CoV-2 are not precisely known. Regarding the eye, more and more studies have pointed out the susceptibility of the conjunctiva to infection with SARS-CoV-2 [6,18,19], but they have not addressed the potential effect of SARS-CoV-2 on the ocular glands. In addition, it is unclear how SARS-CoV-2 induces damage to the ocular surface. In our study, we found, firstly, that the Meibomian glands strongly expressed ACE2, and this was the first time that expression levels of ACE2 in the cornea, the conjunctiva, the Meibomian glands, and the lacrimal glands were compared. We also used co-immunoprecipitation technology to confirm the binding between S protein and ACE2 in the ocular surface. Finally, we proved that the S protein can induce inflammation and cell apoptosis of corneal epithelial cells, conjunctival cells, and Meibomian gland acinar cells. Taken together, our findings indicated that Meibomian glands also expressed ACE2 and that S protein can damage the ocular surface through promoting inflammation and cell apoptosis. However, the lack of direct evidence showing S protein penetrating the cells is a limitation of this study, and this issue should be addressed in future research.

Our studies offer evidence of SARS-CoV-2 transmission through the ocular surface. However, although some clinical studies have confirmed the manifestation of conjunctivitis in COVID-19 patients, the proportion of SARS-CoV-2 RNA detected in conjunctivae is very low. Zhang et al. reported that only 2 patients with conjunctivitis were identified from 72 confirmed COVID-19 patients, and SARS-CoV-2 was found in ocular discharges by RT-PCR in only 1 patient [6]. Another clinical study reported that in 38 patients with COVID-19, 12 patients had ocular manifestations and only 2 patients had positive RT-PCR results after conjunctival swabs [7]. Xia et al. reported that SARS-CoV-2 was detected in conjunctival sacs in only 1 out of 30 patients [22]. In the above studies, conjunctival sac infection rates were very low (1/72, 2/38, and 1/30). Due to the shortcomings of the RT-PCR method, the existence of false negatives could not be ruled out, so the actual positive rate may be slightly higher than these reported values. In addition, the samples collected from conjunctival sacs were limited, so the concentration of the samples might not have been enough for RT-PCR detection of the virus. Another clinical report from Italy showed the Ct values (inversely correlated with viral concentration) detected in late ocular samples were lower than in samples taken five days previously from the same patient [23], suggesting sustained replication in conjunctivae.

To our knowledge, until now, only one clinical study has reported clinical manifestations of the cornea in COVID-19 patients [24]. For one thing, from our results, the expression level of ACE2 in the corneal epithelium is lower than in the conjunctiva. For another, tears and blinking can wash the virus from the cornea to the conjunctival sac. Last but not least, the cornea is an immune-privileged organ. So, the cornea is not prone to clinical symptoms compared to the conjunctiva. Considering the potential risk of corneal infection, it is also vital to pay attention to corneal manifestations in COVID-19 patients.

Our study found that the expression level of ACE2 in the Meibomian gland was 12-fold higher compared to the lung and more than 20-fold higher than in the conjunctiva. However, although our study indicated the potential susceptibility of the Meibomian gland to SARS-CoV-2 infection, no clinical studies have reported the manifestations of the Meibomian gland as yet. We offer three possible explanations: (i) Compared with the conjunctiva, which can be visually observed, Meibomian glands are not easily observed directly. Therefore, they may be ignored by ophthalmologists. (ii) Meibomian glands are protected by the palpebral conjunctiva and eyelid skin, so it is hard for the virus to reach Meibomian gland acinus. (iii) Meibomian gland acinus is continuously breaking down and secreting lipids to form the outer layer of the tear film, so it is possible that this process excretes the virus simultaneously. Some clinical studies have reported the presence of SARS-CoV-2 in tears from confirmed COVID-19 patients [22,25], which may support this perspective. More clinical studies are needed to confirm the effect of SARS-CoV-2 on Meibomian glands.

It has been shown that proinflammatory cytokines and chemokines, including IL-1β, TNF-α, IL-6, interferon gamma-induced protein-10, monocyte chemoattractant protein-1, and macrophage inflammatory proteins 1-α, were significantly elevated in COVID-19 patients [26]. In our study, we found that topical application of the S protein could promote an increase in proinflammatory cytokines in the cornea, the conjunctiva, and the Meibomian glands, with the Meibomian glands being the most prominent.

In summary, our results confirmed the expression of ACE2 in corneal and conjunctival epithelial cells and in Meibomian gland acinar cells, and the S protein could induce damage in these tissues, indicating that the ocular surface epithelium and Meibomian glands could be infected by SARS-CoV-2. But lacrimal glands seem not to be susceptible to SARS-CoV2. When an ophthalmologist comes into close contact with an infected patient during an examination, the saliva of the patient may cause infection in the ophthalmologist through the ocular surface tissue. Therefore, ophthalmologists need to clean their hands after touching a patient’s eyes during an examination to avoid infection by eye–hand–eye contact. From February 26 through 20 March 2020, in the early days of the novel coronavirus epidemic, it was reported that many infections occurred amongst Chinese medical staff, including 14 ophthalmologists, 12 ophthalmic nurses, and 2 ophthalmic technicians, with 3 deaths at Wuhan Central Hospital of China due to occupational exposure [27]. Therefore, more in-depth research into the possibility of ocular transmission of this infectious disease is warranted.

## Data Availability

The data that support the findings of this study are available from the corresponding author, Yang Wu, upon reasonable request.
